# Normal-Hearing Listeners’ and Cochlear Implant Users’ Perception of Pitch Cues in Emotional Speech

**DOI:** 10.1177/0301006615599139

**Published:** 2015-10-18

**Authors:** Steven Gilbers, Christina Fuller, Dicky Gilbers, Mirjam Broersma, Martijn Goudbeek, Rolien Free, Deniz Başkent

**Affiliations:** Center for Language and Cognition Groningen, Department of Applied Linguistics, University of Groningen, The Netherlands; Department of Otorhinolaryngology/Head and Neck Surgery, University Medical Center Groningen, The Netherlands; Center for Language and Cognition Groningen, Department of Dutch, University of Groningen, The Netherlands; Max Planck Institute for Psycholinguistics, Nijmegen, The Netherlands; Centre for Language Studies, Radboud University, Nijmegen, The Netherlands; Department of Communication and Information Sciences, University of Tilburg, The Netherlands; Department of Otorhinolaryngology/Head and Neck Surgery, University Medical Center, Groningen, The Netherlands

**Keywords:** acoustic emotion cues, emotion recognition, cue ranking, cochlear implant, force of articulation

## Abstract

In cochlear implants (CIs), acoustic speech cues, especially for pitch, are delivered in a degraded form. This study’s aim is to assess whether due to degraded pitch cues, normal-hearing listeners and CI users employ different perceptual strategies to recognize vocal emotions, and, if so, how these differ. Voice actors were recorded pronouncing a nonce word in four different emotions: anger, sadness, joy, and relief. These recordings’ pitch cues were phonetically analyzed. The recordings were used to test 20 normal-hearing listeners’ and 20 CI users’ emotion recognition. In congruence with previous studies, high-arousal emotions had a higher mean pitch, wider pitch range, and more dominant pitches than low-arousal emotions. Regarding pitch, speakers did not differentiate emotions based on valence but on arousal. Normal-hearing listeners outperformed CI users in emotion recognition, even when presented with CI simulated stimuli. However, only normal-hearing listeners recognized one particular actor’s emotions worse than the other actors’. The groups behaved differently when presented with similar input, showing that they had to employ differing strategies. Considering the respective speaker’s deviating pronunciation, it appears that for normal-hearing listeners, mean pitch is a more salient cue than pitch range, whereas CI users are biased toward pitch range cues.

## Introduction

In everyday situations, speech not only conveys a message through semantic content but also through indexical cues, such as the talker’s emotional state. The identification of these indexical cues from acoustic stimuli is essential for robust communication in social situations. However, due to the reduced temporal and spectral speech cues in cochlear implants (CIs), the prosthetic hearing devices for sensorineural hearing impaired persons, the users of these devices likely do not make full use of these indexical cues. As a consequence, CI users miss out on an important portion of speech communication, which is perhaps a factor contributing to the difficulties CI users encounter in communicating in noisy environments (Friesen, Shannon, Başkent, & Wang, 2001; Fu & Nogaki, 2005; Fu, Shannon, & Wang, 1998; Hu & Loizou, 2010).

Former studies showed that even in situations without background noise, adult CI users have difficulties recognizing emotions in speech. Adult CI users were shown to recognize emotions in spoken sentences at an accuracy level ranging from 45% to 51% correct only (House, 1994; Luo, Fu, & Galvin, 2007; Pereira, 2000), in contrast to the high accuracy level of 84% to 90% correct in normal-hearing (NH) listeners (House, 1994; Luo et al., 2007). Luo et al. also showed that emotion recognition was better in NH listeners listening to acoustic simulations of CIs (4–8 channels) than in actual CI users. Moreover, these studies suggested that, due to the aforementioned limitations in temporal and spectral cues in CIs, emotion recognition in CI users is mostly based on the acoustic cues of intensity and duration, but not on the cues of pitch or other voice characteristics. Indeed, the representation of the fundamental frequency (*F*_0_) in CIs—and, therefore, pitch perception in CI users—is notoriously degraded (see Moore & Carlyon, 2005 for a review, as well as Gaudrain & Başkent, 2014, 2015 for a discussion on just noticeable differences for voice pitch in CI users and acoustic simulations of CIs). The reduced spectral resolution of the implant is not sufficient to deliver harmonics (in the range of *F*_0_ found in human voices), and therefore, *F*_0_ is generally not perceived strongly through spectral cues. However, as the signal delivered in each electrode is modulated by the speech envelope that carries temporal *F*_0_ cues, pitch perception remains limitedly possible. Studies on gender categorization, another task that relies on the perception of temporal and spectral cues of a speaker’s voice, confirmed that CI users mostly rely on temporal voice pitch cues, whereas NH listeners can utilize both spectral and temporal voice pitch cues (Fu, Chinchilla, & Galvin, 2004; Fu, Chinchilla, Nogaki, & Galvin, 2005; Fuller, Gaudrain, et al., 2014; Kovacic & Balaban, 2009, 2010; Wilkinson, Abdel-Hamid, Galvin, Jiang, & Fu, 2013).

Recently, Massida et al. (2011) pointed at more central factors, such as a cross-modal reorganization of the speech and voice-related areas of the brain that could also affect perception of indexical cues in CI users, in addition to device-imposed limitations. Furthermore, auditory deprivation and subsequent CI use can play an important (negative) role in cognitive processing of perceived speech (Ponton et al., 2000). It has been suggested that acoustically impaired listeners may adapt their perceptual strategies by changing the relative importance of acoustic cues in the perceived speech signal (Francis, Baldwin, & Nusbaum, 2000; Francis, Kaganovich, & Driscoll-Huber, 2008; Fuller, Gaudrain, et al., 2014a; Winn, Chatterjee, & Idsardi, 2011). In other words, the relative importance listeners subconsciously attach to acoustic cues of the perceived speech signal could be determined by the quality of this signal and by which acoustic cues were deemed as more reliable by the listener. Therefore, while pitch perception in CI users has already been shown to be limited and to play a role in reduced emotion recognition in speech, the question still remains whether other factors, such as different processing of the reduced cues to achieve the task, may also play a role.

In this article, we propose an approach to shed further light on this question. More specifically, we propose a method of assessing relative orderings of acoustic emotion cues in terms of salience by ordering them in a way that is reminiscent of the differences in cue weighting for the recognition of phonemes across languages (see e.g., Broersma, 2005, 2010; Fitch, Halwes, Erickson, & Liberman, 1980; Sinnott & Saporita, 2000) and rankings in Optimality Theory (Prince & Smolensky, 1993/2002/2004). These cues are part of the Force of Articulation Model (Gilbers, Jonkers, Van der Scheer, & Feiken, 2013; Van der Scheer, Jonkers, & Gilbers, 2014), which encompasses a wide array of both stereotypical phonetic characteristics of high-arousal speech (e.g., higher pitch and wider pitch range) and more subtle indicators of force of articulation (e.g., number of dominant pitches in a pitch histogram). The advancement this approach brings to the field is that it allows identification of different listener groups’ different biases in auditory perception.

Emotions in speech can be characterized along two dimensions: Valence and Arousal (Fontaine, Scherer, Roesch, & Ellsworth, 2007; Goudbeek & Scherer, 2010; Russell, 1980; Russell & Mehrabian, 1977). The former concerns the difference between positive (e.g., “joy”) and negative emotions (e.g., “sadness”), and the latter concerns the difference between high-arousal (e.g., “anger”) and low-arousal emotions (e.g., “relief”) (see Table 1).

**Table 1.** The Selected Emotions Divided Along the Valence and Arousal Parameters.

		Valence	
		Positive	Negative
Arousal	High	Joy	Anger
	Low	Relief	Sadness

While Luo et al. (2007) did not assess Valence and Arousal, a reinterpretation of their results suggests that with respect to mean pitch and pitch range, speakers only differentiate emotions in their speech along the Arousal parameter. In the present study, we aim to replicate this finding by investigating Valence and Arousal more directly; to that end, we use the four emotions depicted in Table 1, chosen such that Valence and Arousal are fully crossed. Further, we aim to extend this question to a third pitch parameter, namely the number of dominant pitches. On the basis of the findings of Luo et al., we expect that regarding pitch-related force of articulation parameters, speakers only differentiate between emotions along the Arousal parameter and not along the Valence parameter. This expectation is supported by studies on another pitch-related force of articulation characteristic, namely the number of dominant pitches in a pitch histogram, which showed that speech often contains multiple dominant pitches in high-arousal conditions, whereas speech in low-arousal conditions often contains only one dominant pitch (Cook, 2002; Cook, Fujisawa, & Takami, 2004; Gilbers & Van Eerten, 2010; Liberman, 2006; Schreuder, Van Eerten, & Gilbers, 2006).

In this study, in order to investigate whether speakers indeed distinguish between emotions along the Arousal parameter, three pitch-related force of articulation parameters—namely mean pitch, pitch range, and number of dominant pitches—will be acoustically analyzed. Moreover, this study aims to assess which pitch cues are most salient to NH listeners and which ones to CI users. To that end, the aforementioned pitch analyses will also be used to ascertain how individual speakers differ from each other in their production of vocal emotions in nonce words in terms of the degree to which they distinguish between emotions using these pitch cues. Furthermore, this study also assesses listeners’ perception of those cues related to production of the vocal emotions in an emotion recognition experiment. By combining the results of the pitch analyses with the emotion recognition data, we will assess which pitch cues are most salient to NH listeners and which ones to CI users.

In sum, the present study focuses on the production of acoustic emotion cues in speech in a nonce word phrase and on the perception of those cues by NH listeners and CI users. Its main aim is to assess if NH listeners and CI users employ different perceptual strategies to recognize vocal emotions, given that the acoustic cues they can use are not the same, and, if so, how their strategies differ. To this end, an approach to map the two groups’ perceptual strategies for emotion recognition is proposed. This approach builds on Optimality Theory principles and focuses on different acoustic characteristics of force of articulation. Information on individual speakers production of pitch-related acoustic emotion cues is combined with information on NH listeners’ recognition patterns across speakers—both for normal sound and CI simulated sound—and CI users’ recognition patterns across speakers in order to map the two groups’ perceptual biases involved in emotion recognition.

## Methods

### Participants

Twenty NH listeners (17 females, 3 males; ages 19–35 years, *M* = 22.85, *SD* = 3.87) and 20 postlingually deafened CI users (9 females, 11 males; ages 28–78 years, *M* = 65, *SD* = 10.86) with more than 1 year of CI experience participated in the present study. To have a population that represents typical CI users, participants were neither selected based on their device model or their performance with their device nor controlled for age. All participants were native Dutch speakers with no neurological disorders. All NH listeners had pure tone hearing thresholds better than 20 dB hearing level at frequencies of 250 to 4,000 Hz. One CI user was bilaterally implanted. There was one CI user with some residual hearing (only on 250 Hz). This CI user normally wears a hearing aid but did not use this hearing aid during testing. For all other CI users, thresholds were over 60 dB on both ears. Therefore, the other CI users did not have access to any residual hearing that would have interfered with our experiment. Duration of deafness for the CI users ranged from 15 until 23 years. Table 2 shows the demographics of the CI participants.

**Table 2.** CI Participant Demographics.

CI participant	Sex	Age	Duration of CI use	CI type	Processor	Manufacturer
1	M	55	9 years	CI24R CS	CP810	Cochlear Ltd.
2	M	69	4 years	HiRes 90K Helix	Harmony	Advanced Bionics Corp.
3	M	54	3 years	HiRes 90K Helix	Harmony	Advanced Bionics Corp.
4	M	63	1 year	CI24RE CA	CP810	Cochlear Ltd.
5	F	65	4 years	HiRes 90K Helix	Harmony	Advanced Bionics Corp.
6	F	69	11 years	CI24R K	CP810	Cochlear Ltd.
7	M	69	2 years	CI24RE CA	CP810	Cochlear Ltd.
8	M	72	4 years	CI24RE CA	Freedom	Cochlear Ltd.
9	F	78	1 year	CI24RE CA	CP810	Cochlear Ltd.
10	F	67	2 years	CI512	CP810	Cochlear Ltd.
11	M	72	5 years	HiRes 90K Helix	Harmony	Advanced Bionics Corp.
12	M	65	3 years	HiRes 90K Helix	Harmony	Advanced Bionics Corp.
13	F	71	2 years	CI24RE CA	CP810	Cochlear Ltd.
14	M	64	8 years	CI24R CA	CP810	Cochlear Ltd.
15	F	28	10 years	CI24R CS	CP810	Cochlear Ltd.
16	F	62	2 years	CI24RE H	CP810	Cochlear Ltd.
17	M	76	1 year	CI24RE CA	CP810	Cochlear Ltd.
18	F	57	1 year	CI24RE CA	CP810	Cochlear Ltd.
19	F	72	9 years	CI24R CA	Freedom	Cochlear Ltd.
20	M	72	9 years	HiRes 90K Helix	Harmony	Advanced Bionics Corp.

*Note.* CI = cochlear implants.

The present study is a part of a larger project conducted at the University Medical Center Groningen to identify differences in sound, speech, and music perception between NH musicians and nonmusicians, and CI listeners. Therefore, participants largely overlap with the participants in the studies by Fuller, Gaudrain, et al. (2014a)—17 out of 20 of the CI users—and Fuller, Galvin, Maat, Free, & Başkent (2014), and as a result they were experienced with behavioral studies. Further, data from the control group of NH listeners in this study overlap with the data from the nonmusicians in the study by Fuller, Galvin, et al. (2014).

The Medical Ethical Committee of the University Medical Center Groningen approved the study. Detailed information about the study was provided to all participants and written informed consent was obtained before data collection. A financial reimbursement was provided according to the participant reimbursement guidelines of the Otorhinolaryngology Department.

### Stimuli

The recordings used in this study were adjusted from Goudbeek and Broersma’s emotion database (2010a, 2010b). They recorded a nonce word phrase (/nuto hɔm sɛpikɑŋ/) spoken by eight Dutch speakers (four males, four females) for eight different emotions: “joy,” “pride,” “anger,” “fear,” “tenderness,” “relief,” “sadness,” and “irritation” (four takes per emotion). In this study, we used subsets of these stimuli based on pilot tests with NH listeners (Goudbeek and Broersma, 2010a, 2010b). From the original eight emotions, one emotion was selected for each of the four different categories of the Valence-Arousal matrix (Table 1), namely, “joy,” “anger,” “relief,” and “sadness,” which were also the four best recognized emotions on average in the pilot. For pitch analyses, the two best recognized takes for each of the four emotions and for each of the eight speakers were selected. This resulted in a total of 64 tokens (4 emotions × 8 speakers × 2 takes). For the emotion recognition experiment, a further selection was made. The same two takes of the four emotions were used, but only with the four best recognized speakers (two males and two females): Speakers 2, 4, 5, and 6 were selected from the original database for this purpose. This resulted in a total of 32 tokens (4 emotions × 4 speakers × 2 utterances).

### Acoustic Simulation of CI

Similar to studies by Fuller, Gaudrain, et al. (2014) and Fuller, Galvin, et al. (2014), acoustic CI simulations were implemented using sine-wave vocoded simulations based on a Continuously Interleaved Sampling strategy (Wilson, Finley, & Lawson, 1990) with AngelSound Software™ (Emily Shannon Fu Foundation). No distortion was added in the vocoder. Stimuli were first bandlimited by bandpass-filtering (200–7,000 Hz), and then further bandpass-filtered into eight frequency analysis bands (fourth order Butterworth filters with band cutoff frequencies according to the frequency-place formula of Greenwood, 1990). For each channel, a sinusoidal carrier was generated, and the frequency of the sine wave was equal to the center frequency of the analysis filter. The temporal envelope was extracted for each channel through lowpass filtering (fourth order Butterworth filter with cutoff frequency = 160 Hz and envelope filter slope = 24 dB/octave) and half-wave rectification 192. The amplitude of the modulated sine wave was adjusted to match the RMS energy of the filtered signal. Finally, each band’s modulated carriers were summed, and the overall level was adjusted to be equal to that of the original recordings. The motivation for using sine wave instead of noise band excitation was that by doing so, the present study’s results would be directly comparable to the results of previous studies that similarly investigated effects of reduced pitch cues in CIs (e.g., Fu et al., 2004, 2005).

### Procedure

#### Pitch analysis

**Figure 1.**
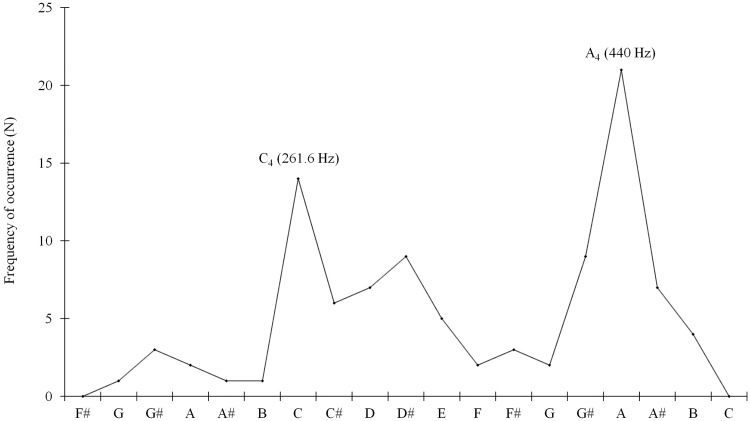
Pitch histogram that shows the semitone frequency for “‘Joy’, speaker 5, take 3” with dominant pitches indicated.

Using PRAAT (version 5.3.16; Boersma & Weenink, 2012) and a PRAAT script designed to measure *F*_0_ (Hz) and intensity (dB) every 10 milliseconds (Cook, 2002), the recordings’ pitch content was analyzed via pitch histograms. Incorrect measurements, for example, when PRAAT mistakenly interpreted higher formants as *F*_0_ or when the increased energy around 5 kHz of the fricatives [s] was interpreted by PRAAT as *F*_0_, were manually removed based on visual inspection of the histograms. All pitch measurements were subsequently rounded off toward the frequency (Hz) of the nearest semitone. Next, the frequency of each semitone per recording was automatically counted for each recording (see Figure 1 for an example of a pitch histogram, which depicts how many times each fundamental frequency—depicted as semitones—occurred in the respective recording and hence does not show any higher harmonics of the individual semitones).

As this study also investigates the differences in perception of emotions in speech, we assessed mean pitch and pitch range in psychoacoustic scales that characterize how people perceive sound (i.e., in Bark and semitones, respectively) rather than in terms of Hertz, the conventional unit of measurement for pitch (Winn et al., 2011): since the ratio of frequencies in Hertz of two notes exactly an octave apart is always 2:1, the difference between an A_2_ note (110 Hz) and an A_3_ (220 Hz) is 110 Hz, whereas the difference between an A_3_ and an A_4_ (440 Hz) is 220 Hz, even though both distances are, according to our auditory perception, exactly the same, namely one octave. For this reason, the pitch measurements in Hertz were first converted from Hertz into Bark and then averaged per recording prior to data analysis. Our motivation for selecting the Bark scale instead of a log frequency scale (which are in fact rather similar to each other) is that the Bark scale is the scale most suitable for analysis of listeners’ perception of formants. Since formants also constitute important acoustic emotion cues we intend to investigate in future studies, selecting the Bark scale for this study already would allow for a better transition into subsequent research (cf. Heeringa, 2004 for a comparison between the Bark scale and frequency scale). The formula used to convert frequencies in Hertz ( *f* ) into Bark was “Critical band rate (Bark) = ((26.81 × *f* )/(1960 + f)) − 0.53.” If the result of this formula was lower than 2, “0.15 × (2 − result)” was added to the earlier result, and if it was higher than 20.1, “0.22 × (result − 20.1)” was added to the earlier result (Traunmüller, 1990). Pitch range for all recordings was first measured in terms of Hertz and then converted into semitones prior to data analysis. The number of dominant pitches was assessed from pitch histograms, such as shown in Figure 1. The acoustic signal was considered to have multiple dominant pitches if aside from the most frequent semitone there was another semitone occurring at least half as frequently as the most frequent semitone. Figure 1 shows two distinct dominant pitches for Speaker 5’s third take of the high-arousal emotion “joy.”

#### Emotion recognition experiment

All participants were tested in an anechoic chamber. The stimuli were presented using AngelSound Software™ (Emily Shannon Fu Foundation) via a Windows computer (Microsoft) with an Asus Virtuoso Audio Device soundcard (ASUSTeK Computer Inc.). After conversion to an analog signal via a DA10 digital-to-analog converter (Lavry Engineering Inc.), the stimuli were played via speakers (Tannoy Precision 8D; Tannoy Ltd.) and were presented at 45 to 80 dB sound pressure level (SPL). No masking noise was used. Participants were seated in an anechoic chamber, facing the speaker at a distance of 1 m, and they registered their responses to stimuli on an A1 AOD 1908 touch screen (GPEG International).

All participants first completed a training series, and then took part in actual data collection. The NH listeners performed the test two times: first with normal acoustic stimuli and second with CI simulated stimuli. CI users performed the test only once. Each test run lasted around 5 minutes. The procedure was the same for training and testing, except for two differences. In training, one condition (normal acoustic stimuli) was tested instead of the full set of 32 tokens. Also, in training, feedback was provided. A thumb was shown on the screen in case of a correct answer, or otherwise, both their incorrect and the correct answers were displayed on screen, and were subsequently also played. Participants were presented one randomly selected stimulus at a time, and stimuli were not presented in blocks per speaker. Per auditory stimulus, the participants’ task was to indicate on the touchscreen monitor which of the four emotions–“anger,” “sadness,” “joy,” or “relief”—they heard. Subsequently, percentage scores according to the number of correctly identified tokens were automatically computed.

CI users were instructed to use their normal volume and sensitivity settings of their devices with no further adjustments during the testing.

#### Ranking of acoustic cues for emotion recognition

To ascertain NH listeners’ and CI users’ emotion cue rankings, the results of the pitch analyses per speaker were compared with the emotion recognition experiment results per speaker for NH listeners and CI users.

### Statistical Analysis

For the statistical analysis, IBM® SPSS® Statistics (version 20) was used. The statistical tests for the pitch analyses were the Mann–Whitney *U* test and the independent samples *t* test, and for the emotion recognition scores the Kruskal–Wallis test and the Mann–Whitney *U* test. A level of *p* < .05 (two-tailed) was considered significant.

## Results

### Pitch Analyses

#### Mean pitch

**Figure 2.**
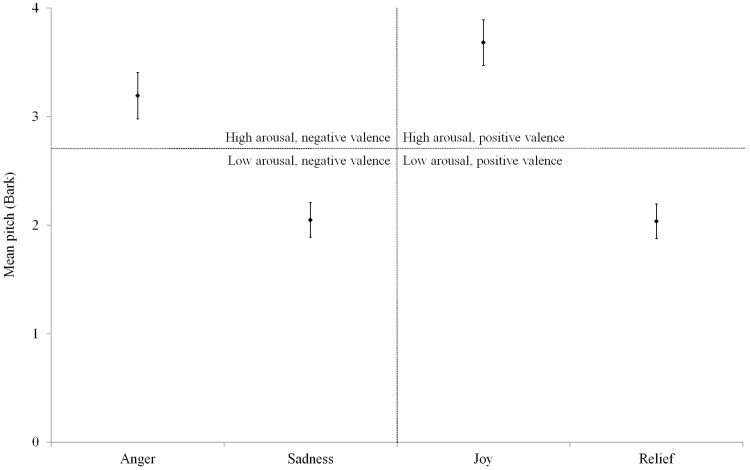
Mean pitch (Bark)—per emotion and with high arousal, low arousal, positive valence, and negative valence indicated; the error bars denote one standard error for this figure and for figures 3, 4, 5, 6, and 7.

Figure 2 shows the mean pitch values (Bark) of the normal acoustic stimuli per emotion with high arousal, low arousal, positive valence, and negative valence indicated. The mean pitch values differed significantly between the four emotions (Kruskal–Wallis test, χ^2^(3) = 30.399, *p* < .001). The mean pitch of high-arousal emotions (“anger” and “joy”) was significantly higher than that of low-arousal emotions (“sadness” and “relief”) (Mann–Whitney *U* test, *U*(*n*_1_ = 32, *n*_2_ = 32) = 111.0, *p* < .001). There was no significant difference regarding mean pitch between positive (“joy” and “relief”) and negative emotions (“anger” and “sadness”; Mann–Whitney *U* test, *U*(*n*_1_ = 32, *n*_2_ = 32) = 449.0, *p* = .398). The mean pitch values differed significantly between the eight speakers (Kruskal–Wallis test, χ^2^(7) = 22.395, *p* < .01). Furthermore, reflecting the observation that female voices are generally perceived as being higher than male voices, female pitch values in Bark were slightly higher than male ones.

#### Pitch range

**Figure 3.**
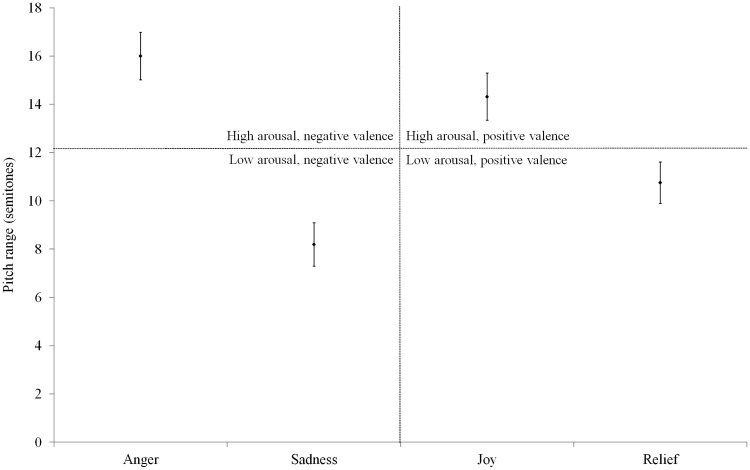
Pitch range (semitones)—per emotion and with high arousal, low arousal, positive valence, and negative valence indicated.

Figure 3 shows the average pitch ranges (semitones) of the normal acoustic stimuli per emotion with high arousal, low arousal, positive valence, and negative valence indicated. The findings were similar to those found for the mean pitch: the pitch range values differed significantly between the four emotions (Kruskal–Wallis test, χ^2^(3) = 28.198, *p* < .001). Further, high-arousal emotions had a significantly wider pitch range in semitones than low-arousal emotions (independent samples *t* test, *t*(62) = 5.944, *p* < .001). No significant difference regarding pitch range was shown between positive and negative emotions (Mann–Whitney *U* test, *t*(62) = −.365, *p* = .716). Furthermore, the pitch range values differed significantly between the eight speakers (Kruskal–Wallis test, χ^2^(7) = 21.217, *p* < .01).

#### Dominant pitches

**Figure 4.**
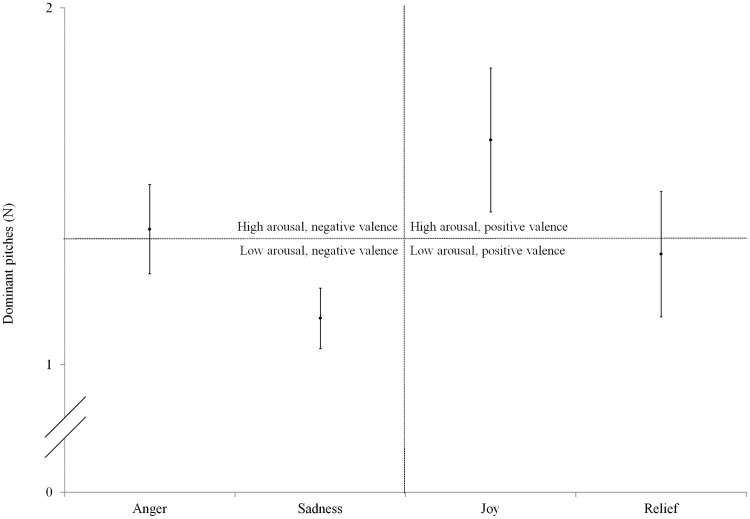
Number of dominant pitches—per emotion with high arousal, low arousal, positive valence, and negative valence indicated.

Figure 4 shows the number of dominant pitches in the normal acoustic stimuli, averaged per emotion with high arousal, low arousal, positive valence, and negative valence indicated. In contrast to the findings for mean pitch and pitch range, the number of dominant pitches did not differ significantly between the four emotions (Kruskal–Wallis test, χ^2^(3) = 6.102, *p* = .107). The number of dominant pitches was significantly higher for high-arousal emotions than for low-arousal emotions (Mann–Whitney *U* test, *U*(*n*_1_ = 32, *n*_2_ = 32) = 378.5, *p* < .001). Positive and negative emotions did not differ significantly regarding the number of dominant pitches (Mann–Whitney *U* test, *U*(*n*_1_ = 32, *n*_2_ = 32) = 452.0, *p* = .313). No significant difference was found among the eight speakers regarding the number of dominant pitches (Kruskal–Wallis test, χ^2^(7) = 13.687, *p* = .057).

### Emotion Recognition Experiment

**Figure 5.**
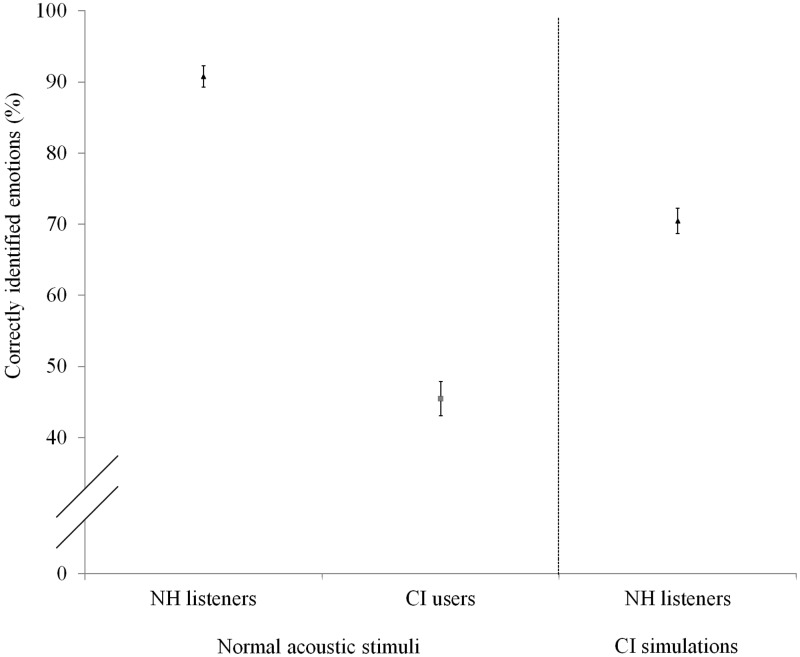
Percentage of correctly identified emotions per condition (normal acoustic stimuli on the left, CI simulations on the right).

Figure 5 shows the mean emotion recognition for NH participants, for both the normal acoustic stimuli and the CI simulations, as well as for the CI users (with normal acoustic stimuli only). It should be noted here that for the emotion recognition experiment, performance at chance level corresponds to 25% of the emotions being correctly identified. NH participants significantly outperformed CI users with regard to emotion recognition for the normal acoustic stimuli (Mann–Whitney *U* test, *U*(*n*_1_ = 20, *n*_2_ = 20) = .000, *p* < .001). The NH listeners listening to CI simulations also scored significantly better than the CI users (independent samples *t* test, *t*(38) = 6.888, *p* < .001).

#### NH and CI emotion recognition patterns

**Figure 6.**
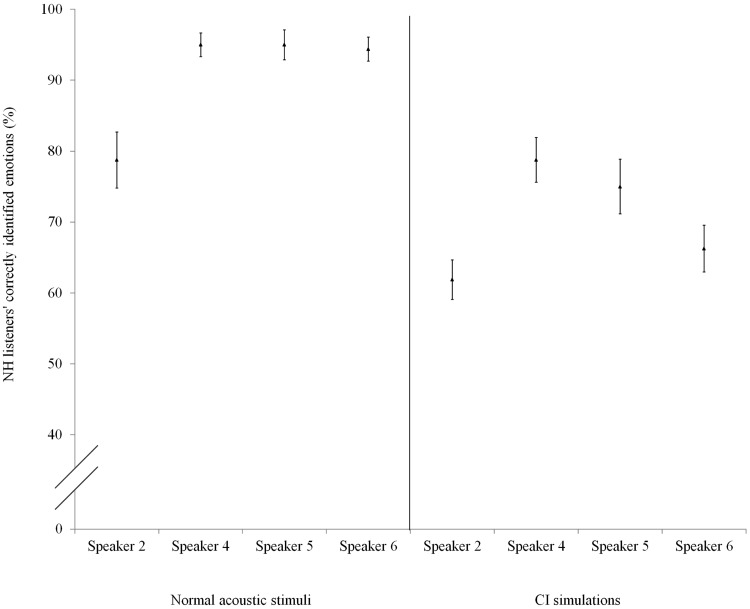
NH listeners’ percentage of correctly identified emotions per speaker (normal acoustic stimuli on the left, CI simulations on the right); Speaker 2 and Speaker 4 are male, Speaker 5 and Speaker 6 are female.

Figure 6 shows the recognition of the emotions per speaker for the NH listeners. The recognition of the four speakers’ emotions differed significantly for normal acoustic stimuli (Kruskal–Wallis test, χ^2^(3) = 18.343, *p* < .001) as well as for CI simulations (Kruskal–Wallis test, χ^2^(3) = 13.527, *p* < .01). Post hoc tests show that NH listeners recognized Speaker 2’s emotions significantly worse than all other speakers’ emotions for the normal acoustic stimuli and significantly worse than Speaker 4’s in the CI simulations (Table 3).

**Table 3.** Significantly Different Speaker Pairs (Bonferroni corrected) for NH Listeners According to a Post Hoc Analysis Using the Mann–Whitney *U* Test.

Condition	Worse speaker	Better speaker	Significance
Emotion	Speaker 2	Speaker 4	*p* < .01
		Speaker 5	*p* < .01
		Speaker 6	*p* < .01
CI simulations	Speaker 2	Speaker 4	*p* < .01

*Note.* CI = cochlear implants.

**Figure 7.**
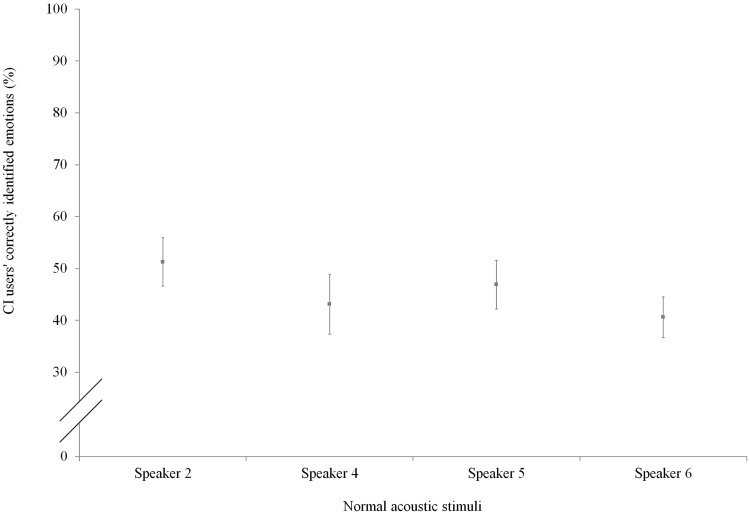
CI users’ percentage of correctly identified emotions per speaker (normal acoustic stimuli).

Figure 7 shows the recognition of the emotions per speaker for the CI users. Contrary to the NH listeners’ recognition scores, the CI users’ recognition scores of the four speakers did not differ significantly (Kruskal–Wallis test, χ^2^(3) = 2.977, *p* = .395).

## Discussion

### Methodology

The present study’s aim was to create a framework that accounts for the relative weights of different acoustic pitch cues regarding emotion perception in speech. The method deviates from previous emotion recognition studies in NH listeners and CI users (e.g., Luo et al., 2007). Firstly, this study is based on a nonce word phrase, which is devoid of any meaning, as opposed to real-language sentences for which it is arguably harder to control whether they are completely semantically neutral. As a result, the possibility that any semantic content of the stimuli would influence participants’ emotion recognition could be safely ruled out in the present study. Furthermore, as this study investigates the differences in perception of emotions in speech, we assessed mean pitch and pitch range in psychoacoustic scales that accurately represent how people perceive sound (i.e., in Bark and semitones, respectively) rather than in terms of Hertz, the usual unit of measurement for pitch (Winn et al., 2011), which was used in previous emotion recognition studies (see e.g., Luo et al., 2007). In addition, the present study investigated a pitch parameter from the Force of Articulation Model (Gilbers & Van Eerten, 2010; Gilbers et al., 2013) that previous CI emotion recognition studies did not, namely the number of dominant pitches occurring in different vocal emotions.

### Pitch Analyses

The present study’s results support its expectations regarding mean pitch (higher mean pitch for high-arousal than low-arousal emotions), pitch range (wider pitch range for high-arousal than low-arousal emotions), and number of dominant pitches (more dominant pitches for high-arousal than low-arousal emotions). The results for mean pitch and pitch range confirm Luo et al.’s (2007) conclusions, and the results for number of dominant pitches confirm the conclusions of Cook (2002), Cook et al. (2004), Schreuder et al. (2006), Liberman (2006), and finally Gilbers and Van Eerten (2010). These studies claim high-arousal speech to be characterized by significantly more frequency peaks, that is, dominant pitches, than low-arousal speech. In other words, the number of dominant pitches is a cue for the level of arousal in speech. The results also validate our decision to use the arousal-based force of articulation parameters for the assessment of NH listeners’ and CI users’ emotion cue rankings. In addition, the results show that regarding pitch-related emotion cues, speakers differentiate between emotions along the Arousal parameter but not along the Valence parameter.

### Emotion Recognition in NH Listeners and CI Users

NH listeners were shown to outperform CI users with regard to emotion recognition for the normal acoustic stimuli and also when listening to CI simulations. Please note, however, that because the selected four emotions were the best recognized by NH listeners in the pilot tests, the NH participants might have had an extra advantage compared with the CI users during the emotion recognition experiment. The results can, as a result, not be generalized to all vocal emotions; they are limited to the vocal emotions selected for this study. Moreover, with respect to the role of duration and amplitude cues, it should be noted that when the emotion recognition experiment was conducted, NH listeners and CI users were also tested in conditions with stimuli normalized for duration (1.77 seconds) and amplitude (65 dB SPL). However, since both groups’ emotion recognition scores for these conditions were extremely similar to those of the non-normalized conditions (the differences were insignificant across the board), we chose not to include them in our manuscript for reasons of brevity.

Moreover, both groups’ recognition scores were compared across speakers to assess whether any speakers’ emotions were recognized better or worse than other speakers’—information which could be employed to ascertain NH listeners’ and CI users’ possibly differing perceptual strategies. In this respect, it was found that Speaker 2’s emotions were notably recognized worse in comparison to the other speakers’ by NH listeners but not by CI users. Even when presented with similar stimuli (CI simulated stimuli for NH listeners and actual CI sound stimuli for CI users), the degree to which emotions were correctly identified differed across speakers for NH listeners (with Speaker 2’s emotions being the worst recognized), but it did not for CI users, who recognized each speaker’s emotions equally well. In this respect, it should be noted that complete informational equivalence of the stimuli cannot be assumed when comparing emotion recognition in CI simulated stimuli with recognition in actual CI sound stimuli, as simulated CI sound is an approximation of actual CI sound. Nevertheless, the results seem to indicate a difference in strategies for perceiving emotions in speech between NH and CI listeners, as was suggested by Winn et al. (2011). The CI users’ differing perceptual strategy is likely due to the degraded cues transmitted with the CI device but also possibly due to long-term loss of hearing leading to neuroplasticity and exposure to CI-processed sound leading to adaptation. CI users may, therefore, differ from NH listeners in the relative value they attach to certain emotion cues (e.g., pitch range and mean pitch). In short, due to CI devices’ technical limitations and the long-term hearing loss with possible loss of neuronal tissue, many acoustic cues are more difficult to perceive for CI users than for NH listeners. Since the NH listeners never had to adjust their perceptual strategies according to CI-like input, they made use of their regular perceptual strategies even in the simulated testing condition, which, as evident from the fact that they recognized one speaker’s emotions less often than the others, was not optimal for the CI-simulated input.

Once the difference in perceptual strategies between NH listeners and CI users was established, we analyzed how these differed. Our results indicate that acoustic cues for emotion recognition are ranked by listeners on the basis of salience, and that these cue orderings are different for NH listeners and CI users. This hierarchy is reminiscent of how cues are weighted differently for the recognition of phonemes across languages. Languages differ in the use of perceptual cues for phonetic contrasts. The weights that listeners from different language backgrounds assign to the same cues and for very similar phoneme contrasts can differ strongly, and when listening to a second language, listeners often find it difficult to weigh the cues appropriately, as they tend to pay attention to cues that are important in their own language, and disregard cues that are crucial in the second language (Broersma, 2005, 2010). The acoustic emotion cue hierarchy is also reminiscent of how constraints are ranked differently for different languages within the linguistic framework of Optimality Theory (Prince & Smolensky, 1993/2002/2004). Optimality Theory is a nonderivational linguistic theory in which constraints on outputs determine grammaticality. All constraints are universal and available in every language, but languages differ in the way the constraints are ranked in a language-specific dominance hierarchy. In other words, certain constraints are more important in one language than in another. In a similar way, we hypothesize that all acoustic emotion cues are universal and available to all listeners, although due to the limitations in sound transmission in the electrode-nerve interface, cues such as static *F*_0_ levels (mean pitch) are more difficult to discern for CI users than for NH listeners—low *F*_0_ levels are difficult to detect for CI users, but they can still deduce pitch information from the temporal envelope in the signal—and hence CI users have ranked these cues lower in their perceptual strategy than possibly more easily discernible, dynamic *F*_0_ cues (e.g., pitch range). In contrast, since mean pitch is a very robust cue in the acoustic signal, NH listeners, who can easily discern it, place this cue highly on the acoustic emotion cue dominance hierarchy. In the following section, it will be discussed whether we can find evidence for how exactly these cue rankings differ for both groups.

#### Preliminary analysis of cue orderings

In our new approach to acoustic emotion recognition in NH listeners and CI users, which involves the assessment of acoustic emotion cues’ relative salience to listeners, two types of information need to be combined in order to ascertain cue rankings: How individual speakers differ from each other in terms of how strongly they distinguish between emotions using pitch, and how they differ from each other concerning how well their emotions are recognized by NH listeners and by CI users. Suppose one particular speaker deviates from the others, for example, because this speaker contrasts between high and low-arousal emotions more extremely regarding mean pitch. If this speaker’s emotions were recognized better than the other speakers’ by NH listeners but not by CI users, this would indicate that mean pitch is a relatively important pitch cue for NH listeners and a relatively unimportant one for CI users.

Preliminary analysis of the difference between Speaker 2 (the speaker whose vocal emotions were recognized worse than the other speakers’ by NH listeners) and the other speakers concerning mean pitch indicates that unlike the other speakers, Speaker 2’s mean pitch is below the lowest fundamental frequency one can perceive with a CI device (160 Hz). The fundamental frequency can be perceived with a CI, but only weakly from the temporal envelope cues. This might suggest that mean pitch is relatively less important for CI users since they did not recognize Speaker 2’s emotions any worse than the other speakers.

Regarding pitch range, Speaker 2’s high–low arousal emotions ratio is more extreme than the other speakers' (roughly 3:1 and 2:1, respectively), and since Speaker 2’s emotions were among the better recognized for CI users (note that this difference was not significant) and were the worst recognized by NH listeners (note that this difference was significant), this seems to indicate that CI users attach relatively more value to how much a speaker distinguishes between high- and low-arousal emotions in terms of pitch range than NH listeners do.

On the basis of these findings, we hypothesize that for CI users, pitch range is a relatively more salient acoustic emotion cue than mean pitch and is thus ranked higher than mean pitch in their perceptual strategy. In contrast, we hypothesize that for NH listeners, mean pitch is a more salient cue than pitch range, and hence that the former is ranked higher than the latter. Since no speakers deviated from their peers’ speech production with respect to the number of dominant pitches, it is thus far not possible to assess the relative rankings of this cue for NH listeners and CI users.

### Suggestions for Future Research

The present study has made use of the speech emotion database recorded by Goudbeek and Broersma (2010a, 2010b), which is based on recordings of the nonce word phrase /nuto hɔm sɛpikɑŋ/. This database already allows for analysis of certain important force of articulation parameters such as speech rate, mean pitch, pitch range, segment duration, syllable isochrony, and (lack of) vowel reduction. The Force of Articulation Model (Gilbers et al., 2013), however, consists of other parameters as well, and the nonce word phrase /nuto hɔm sɛpikɑŋ/ does not contain segmental content required for these parameters to be measured. For instance, force of articulation also manifests itself in plosives (in the relatively long duration of their release burst, their occlusion, and their voice onset time), liquids (in the relatively high F_2_ and low F_1_ of /l/), and the extreme vowels /a,i,u/ (in which an expansion of the vowel space can be measured if /a,i,u/ occur in comparable, e.g., stressed, positions). Since not all of these segments are present in the nonce word phrase /nuto hɔm sɛpikɑŋ/, the current database does not allow for analysis of all Force of Articulation Model parameters. Therefore, to assess more extensive emotion cue rankings for CI users and NH listeners than presented in this study, an extended speech emotion database that also allows for the analysis of such additional Force of Articulation Model parameters needs to be recorded.

Furthermore, the present study’s results show that regarding pitch-related emotion cues, speakers differentiate between emotions not along the Valence parameter but along the Arousal parameter. However, it remains unclear how they differentiate between positive and negative emotions, and future research needs to be conducted to see which cues play a role in making this distinction.

## Conclusion

The present study proposes an approach to answer the question whether, and, if so, how CI users and NH listeners differ from each other regarding their perceptual strategies in processing of speech emotions, namely by combining results from phonetic analyses of emotional speech to the results of emotion recognition experiments. The results of the study’s pitch analyses and emotion recognition experiment confirm the hypotheses. The fact that CI users’ and NH listeners’ emotion recognition patterns were significantly different indicates that their perceptual strategies in identifying emotional speech may indeed be different for CI users than for NH listeners. This is likely the result of some acoustic cues being only partially available to CI users and of different degrees of cue sensitivity for CI users and NH listeners. NH listeners’ and CI users’ relative rankings of the number of dominant pitches cue could not be assessed yet, for all speakers performed similarly regarding this cue. However, it appears that NH listeners and CI users differ from each other regarding which acoustic cues of emotional speech they find more salient. This idea is supported by preliminary analyses suggesting that for NH listeners, mean pitch is a more salient cue than pitch range, whereas CI users are biased toward pitch range cues.
